# Chronic hypokalemia due to excessive cola consumption: a case report

**DOI:** 10.1186/1757-1626-1-32

**Published:** 2008-07-14

**Authors:** Clifford D Packer

**Affiliations:** 1 Louis Stokes Cleveland Veterans Affairs Medical Center, 10701 East Boulevard, Cleveland, Ohio, 44106, USA

## Abstract

A 52-year-old man was noted to have severe chronic hypokalemia despite discontinuation of diuretic treatment for hypertension and aggressive oral potassium supplementation. His serum potassium normalized temporarily when he was hospitalized, but hypokalemia recurred after discharge. He complained of generalized weakness and fatigue, and occasional loose stools. Physical examination showed mild generalized muscle weakness. Laboratory testing ruled out renal potassium wasting. A dietary history revealed that he was consuming 4 liters of cola per day, with a calculated fructose load of 396 grams per day. Since fructose absorption in the small bowel is relatively inefficient, this probably led to an osmotic diarrhea and GI potassium wasting. Physicians should ask their patients about soft drink consumption when they encounter unexplained hypokalemia.

## Introduction

There are 4 published case reports of severe chronic hypokalemia due to long-term, excessive cola consumption. [1-4] Complications described in the reports include hypokalemic myopathy, hypokalemic nephropathy, and nephrogenic diabetes insipidus. In this case, a patient developed severe chronic hypokalemia and probable hypokalemic myopathy due to consumption of 4 liters of Pepsi-Cola per day.

## Case Report

**Figure 1:**
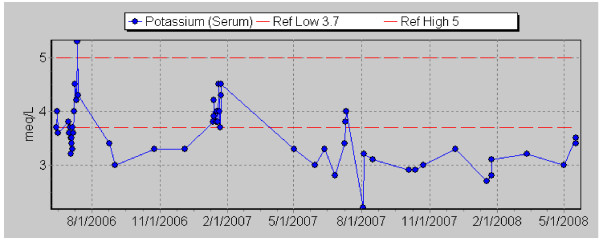
**Serum Potassium Values from July 2006 through May 2008**. Note normalization of serum potassium levels during hospitalizations in 7/06, 1/07, and 7/07. Also note improvement in potassium level from 3.0 to 3.5 mg/dL between 5/1/08 and 5/16/08, when the patient decreased his cola consumption from 4 liters to 2 liters per day.

A 52-year-old white male with O2-dependent COPD, hypertension, GERD, idiopathic gastroparesis, and chronic low back pain was noted to have persistent hypokalemia in the 2.7–3.3 meq/L range over more than 2 years. He complained also of chronic generalized weakness and fatigue. He denied nausea or vomiting, but did have occasional loose stools. The hypokalemia persisted despite discontinuation of diuretic treatment for hypertension and fludrocortisone that had been prescribed briefly for orthostatic hypotension. There was no improvement with aggressive oral potassium supplementation in amounts up to 120 meq per day. The patient's serum potassium level normalized on three occasions when he was hospitalized and given supplemental potassium (COPD exacerbations in 7/06 and 1/07, pseudoseizures in 7/07), but the hypokalemia promptly recurred after discharge from the hospital (Figure 1). His medications were paroxetine, trazodone, pregabalin, sustained-release morphine, loratadine, isosorbide mononitrate, lisinopril, metoprolol, simvastatin, omeprazole, metoclopramide, potassium chloride, calcium/vitamin D tablets, alendronate, and mometasone, tiotropium, and albuterol inhalers. He smoked one-half pack of cigarettes per day and did not drink alcohol.

On physical examination, he was a chronically ill-appearing man wearing a nasal cannula. Height was 69 inches, weight 205 pounds. There were no cushingoid facies, buffalo hump, or abdominal striae. Vital signs were temperature 98.6 degrees, pulse 95, respiratory rate 14, blood pressure 128/73. There was no thyromegaly or lymphadenopathy. Lungs showed decreased breath sounds and mild expiratory wheezes bilaterally. Heart sounds were regular with no murmurs, rubs, or gallops. The abdomen was soft and non-tender, with no masses or organomegaly. Extremities showed no edema, clubbing or cyanosis. The neurologic examination revealed mild generalized muscular weakness (4+/5) and normal deep tendon reflexes.

Laboratory results include serum sodium 137 mg/dL, potassium 3.0 mg/dL, chloride 95 mmol/L, CO2 30.0 mmol/L, blood urea nitrogen 5 mg/dL, creatinine 0.8 mg/dL, calcium 9.3 mg/dL, phosphorus 4.1 mg/dL, albumin 3.6 g/dL, ferritin 126 ng/mL, hemoglobin 12.7 g/dL, white blood cell count 10.6 K/cmm, and platelet count 160 K/cmm. Serum aldosterone was 4.8 ng/dL (normal 4–31 ng/dL) and the plasma renin activity was 0.33 ng/mL/hr (normal 1.31–3.96 ng/mL/hr upright, 0.15–2.33 ng/mL/hr supine). Spot urine potassium was 8.6 mEq/L, urine sodium was < 10 mEq/L, and urine chloride was 16 mmol/L.

In the absence of a clear explanation for this patient's chronic hypokalemia, he was asked to give the details of his diet. He admitted to drinking 4 liters of Pepsi-Cola per day for the past several years. It was his habit to sip cola slowly but almost continuously, throughout the day. When hospitalized, he had stopped drinking cola and his potassium levels had temporarily normalized. In early May 2008, he decreased his cola intake to 2 liters per day, with a resultant increase in the serum potassium from 3.0 to 3.5 mg/dL (Figure 1).

## Discussion

The normal plasma renin activity, normal serum aldosterone, and low urine potassium suggest that this patient's hypokalemia was not caused by renal potassium wasting. The problem persisted after discontinuation of hydrochlorothiazide, which also eliminates diuretic-induced hypokalemia as a possible cause. This strongly suggests that the chief problem was either gastrointestinal potassium loss, inadequate potassium intake, or some combination of the two.

The main ingredients of Pepsi-Cola [5] are high-fructose corn syrup, sugar, colorings, phosphoric acid, caffeine, citric acid, and natural flavors. It contains 9.8 mg/liter of sodium and 42.3 mg/liter of potassium. There are 110.4 g/liter of high-fructose corn syrup in Pepsi-Cola, so it follows that this patient was consuming approximately 440 grams of high-fructose corn syrup per day. High-fructose corn syrup is 90% fructose and 10% glucose, which calculates to a daily fructose intake of 396 grams. [6] Fructose is absorbed in limited quantities (only about 40% as compared with glucose) by a facilitated transport mechanism in the small intestine. [7] Therefore, a large amount of unabsorbed fructose passed into the colon, causing an osmotic diarrhea and chronic potassium depletion. Often, because of his gastroparesis, the patient's daily caloric intake consisted mostly (if not entirely) of cola with high-fructose corn syrup and minimal potassium. Four liters of Pepsi-Cola contains only 169 mg of potassium, less than 5% of the recommended daily allowance of 3.5 grams, so inadequate dietary potassium probably played a role as well.

## Conclusion

Excessive soft drink consumption can cause hypokalemia due to a fructose-induced osmotic diarrhea. Given the very high soft drink consumption in industrialized societies, this is probably an underreported and underdiagnosed cause of potassium depletion. In addition to muscle weakness and cramping, hypokalemia lowers the arrhythmia threshold and may increase the risk of sudden death, particularly in people with heart disease. Physicians should ask their patients about soft drink consumption when faced with unexplained hypokalemia.

## Abbreviations

COPD: Chronic obstructive pulmonary disease; GERD: Gastroesophageal refux disease.

## Consent

Written informed consent was obtained from the patient for publication of this case report and accompanying images. A copy of the written consent is available for review by the Editor-in-Chief of this journal.

## Competing interests

The author declares that they have no competing interests.

## Authors' contributions

CDP cared for the patient, collected and interpreted the laboratory data, researched the patient's medical condition, and was the sole author of the manuscript.
